# Exercise-Based Cardiac Rehabilitation Improves Left Ventricular Dysfunction, Mitophagy, and Oxidative Stress Postmyocardial Infarction

**DOI:** 10.1155/crp/7778063

**Published:** 2025-07-23

**Authors:** Changyong Wu, Haojie Li, Shuangfeng Zhao, Jiang Liu, Ruijie Li, Huang Sun, Suli Bao, Menghan Li, Yunzhu Peng

**Affiliations:** ^1^Department of Cardiology, The First Affiliated Hospital of Kunming Medical University, Kunming, China; ^2^Department of Cardiology, Xiangyun County People's Hospital, Dali, China; ^3^Department of Cardiology, Fuwai Yunnan Cardiovascular Hospital, Kunming, China; ^4^Department of Reproduction and Genetics, The First Affiliated Hospital of Kunming Medical University, Kunming, China; ^5^Kunming Medical University, Kunming, China; ^6^Department of Cardiology, The Affiliated Hospital of Yunnan University, Kunming, China

**Keywords:** cardiac fibrosis, cardiac rehabilitation, exercise training, mitophagy, myocardial infarction, oxidative stress

## Abstract

**Aim:** Left ventricular dysfunction, disturbed mitophagy, and persistent oxidative stress after myocardial infarction (MI) are critical drivers of myocardial injury and cardiac remodeling. Exercise-based cardiac rehabilitation (CR) is a cornerstone of post-MI treatment and management, yet its mechanistic effects on myocardial repair remain incompletely elucidated. This study aimed to the effect of exercise-based CR on the left ventricular dysfunction, mitophagy, and oxidative stress post-MI.

**Methods:** Mendelian randomization analysis elucidated causal relationship between six physical activities and MI. Subsequently, 70 MI patients were randomized to control or exercise-based CR groups (moderate-to-vigorous physical activity intensity, 3 days/week, 10–50 min/day, 12 weeks); left ventricular function, cardiopulmonary function, and SF-36 quality of life scale were assessed pre-/postintervention using standardized protocols. Additionally, 21 rats were allocated to Sham, MI, or MI + treadmill running groups (high-intensity interval exercise training, 5 days/week, 30–50 min/day, 10–25 m/min, 4 weeks); left ventricular function, mitophagy, and oxidative stress were detected postintervention.

**Results:** Genetically predicted moderate-to-vigorous intensity physical activity was significantly associated with lower risk of MI (IVW OR = 0.66, 95% CI: 0.54–0.81), with no causal links for other activities. Critically, clinical and animal studies demonstrated that exercise-based CR improved left ventricular systolic function (LVEF) after MI. Four-week exercise in MI rats enhanced mitophagy levels (LC3, FUNDC1, PINK1, and Parkin) and attenuated oxidative injury (MDA, GSH, SOD2, and GPX4) post-MI. Additionally, exercise-based CR also improved cardiopulmonary function (peak VO_2_/kg, peakVO_2_/pred%, and MET) in patients with MI and ameliorated mitochondrial damage in MI rats. However, GLS, secondary cardiopulmonary parameters (Wmax, HRR1min, peakVO_2_/HR, and peakVO_2_/HRpred%), and SF-36 (PCS and MCS) showed no significant changes, which may be associated with shorter duration of exercise intervention.

**Conclusion:** Exercise-based CR significantly ameliorated left ventricular dysfunction, enhanced mitophagy levels, and attenuated oxidative stress post-MI, establishing its role in critical pathological mechanisms. Future studies should validate long-term sustainability of exercise-based CR and explore the interaction mechanism between mitophagy and oxidative stress in cardiac remodeling, providing personalized and precise exercise protocols for people at high risk of exercise.

## 1. Introduction

Myocardial infarction (MI) remains the leading cause of cardiovascular death worldwide, characterized by high prevalence, high mortality, multisystem complications, and poor prognosis, significantly exacerbating the burden of global health care resources [1]. Despite current reperfusion therapy, drug therapy, and device intervention have improved the survival rate, post-MI heart failure prevalence continues to rise, reflecting unmet needs in long-term myocardial repair. Exercise training, as a central element of cardiac rehabilitation (CR), is recommended as a nondrug therapy that effectively mitigates residual risk factors, reduces hospitalization and mortality, and improves function of multiple organs, finally establishing its role as a cornerstone of integrated lifecycle management post-MI [2, 3].

Increasing evidence supported implementing exercise training during the acute MI phase alongside standard treatment [2, 4, 5]. Studies have shown that exercise training intensity was associated with cardiovascular disease prognosis. For example, compared with moderate-intensity continuous training (MICT) and routine physical activity, high-intensity interval training (HIIT) superiorly improved cardiopulmonary function and exercise capacity in post-MI patients with good safety and feasibility [6, 7]. Additionally, previous studies have identified the protective effects of exercised-based CR in cardiovascular diseases, including reducing the inflammatory response and oxidative stress, improving microvascular dysfunction, and promoting cardiac metabolism [8]. However, the molecular mechanism of HIIT has not been elucidated, and there remains a lack of effective biomarkers to monitor the efficacy and prognosis of CR.

Cardiac pressure or volume overload and excessive synthesis of cytokines, growth factors, and chemokines promote the proliferation and activation of myocardial fibroblasts after MI, resulting in excessive deposition of the extracellular matrix, thereby aggravating cardiac dysfunction and promoting heart failure formation [9]. Inflammatory infiltration, dysfunction of mitochondrial respiratory chain complexes, and decreased antioxidant enzyme activity following myocardial injury promote reactive oxygen species (ROS) production, resulting in mitochondrial structural and function impairment. However, mitophagy is an important physiological process and defense mechanism, mainly transporting damaged and senescent mitochondria to lysosomes for degradation and recycling [10]. Therefore, regulating mitophagy levels becomes a potential therapeutic strategy for cardiovascular health, particularly PINK1/Parkin or FUN14 domain-containing protein 1 (FUNDC1)-mediated mitophagy. Few studies have focused on the effect of mitochondrial oxidative damage and mitophagy in myocardial fibrogenesis [11]. This study aimed to investigate HIIT-mediated cardioprotection in left ventricular dysfunction, mitophagy, and oxidative stress and explored the possible interaction molecular mechanism between mitophagy and oxidative stress in myocardial fibrosis.

## 2. Materials and Methods

### 2.1. Two-Sample Mendelian Randomization (TSMR) Analysis

The study design was shown in Figure S1. The summary statistical data were extracted from large scale GWAS studies for sleep duration (SD) [12], sedentary-to-activity transition probability (SATP) [13], light-intensity physical activity (LPA) [13], moderate-intensity physical activity (MPA) [12], moderate-to-vigorous intensity physical activity (MVPA) [14], and vigorous-intensity physical activity (VPA) [15]. GWAS summary statistics for MI (14,825 cases and 380,970 controls) are extracted from the Coronary Artery Disease Genome-Wide Replication and Meta-analysis plus the Coronary Artery Disease Genetics (CARDIoGRAMplusC4D) consortium [16]. The detailed information of GWAS data was in Table S1. We extracted single nucleotide polymorphisms (SNPs) robustly at the genome-wide significant level (*p* < 5 × 10^−7^) and pruned all SNPs with the stringent pairwise linkage disequilibrium (LD) *r*^2^ < 0.001 and clumping distance > 10,000 kb. Additionally, each selected instrument SNP was examined for potential violations of the assumptions 2 and 3 based on the online platform LDtrait tool (https://ldlink.nih.gov/). F-statistic greater than 10 indicates a strong association. The related genetic variants can be seen in Table S2.

The flowchart of TSMR analysis was presented in Figure S2. First, the pleiotropic outliers were identified using MR pleiotropy residual sum and outlier (MR-PRESSO) method and removed before conducting MR analysis. And the MR-Egger was used to identify potential directional pleiotropy, where *p* > 0.05 for intercept that illustrated no evidence of horizontal pleiotropy. Second, heterogeneity was evaluated by Cochran's *Q*-statistics and the I^2^ test. With a *p* < 0.05 indicating the presence of heterogeneity, consequently, a random-effect inverse variance weighted (IVW) method would be used. Third, the causal effects were estimated by the IVW method. We also performed weighted median (WM) and MR-Egger to assess the robustness of the results because the IVW method requires all the IVs to be valid in order to obtain an unbiased estimate. If the results obtained by these three MR-based analytic methods were all in the same direction, the results of MR analysis were considered to be consistent. Finally, the leave-one-out method was implemented by sequentially excluding each SNP to determine whether the estimates were driven by any single SNP.

### 2.2. Study Subjects

Between April 2022 and August 2023, all patients with MI after percutaneous coronary intervention (PCI) at our center were screened for inclusion. This study was approved by the ethics committee of the First Affiliated Hospital of Kunming Medical University, and the number is (2022) Ethics L No. 256. Subjects were included if they fulfilled the following: 1 month after PCI, aged between 18 and 75 years, low and moderate risk of exercise, and provided written informed consent. The exclusion criteria included exercise-limited diseases mainly including orthopedic disorders, server neurologic conditions, and thromboembolism; other cardiovascular diseases such as decompensated heart failure, moderate-server valvular heart disease, and acute myocarditis; complicated with sever systemic disorder such as hepatic and renal failure; sever arrhythmias such as irreversible atrial and ventricular arrhythmias, atrio ventricular bloc, needing implantation of a pacemaker or implantable cardioverter defibrillators; participating in or planning to other intervention studies within 3 months; and currently receiving systematic training. The coin was used to assign participants in a 1:1 ratio to the control group and the exercise group. Clinical data were collected, including demographic characteristics, medical history, physical examination, laboratory parameters, echocardiography, cardiopulmonary exercise test (CPET) indices, and 36-item short form health survey (SF-36).

All animal experimental procedures were conducted in accordance with the Guide for Care and Use of Laboratory Animals (United States National Institutes of Health, 8^th^ Edition, 2011) and were approved by the Institutional Animal Use and Care Committee of Kunming Medical University (Kunming, China). The approval number is kmmu20221866. 24 male Sprague–Dawley rats (200 ± 20 g) were purchased from the Laboratory Animal Department of Kunming Medical University. All rats were raised in a humidity/temperature-controlled pathogen-free environment with a light-12 h/dark-12 h cycle, with food and water ad libitum. The MI model was established by ligation of the left anterior descending coronary artery (LAD) as previously described [17, 18]. In brief, the rat was anesthetized with sodium pentobarbital and fixed. The chest was opened to expose to the heart. The LAD was ligated with a 6-0 silk suture at 2-3 mm distal to the pulmonary artery conus beneath the left coronary venous trunk. Successful modeling was confirmed by visual observation of pallor or whitening in the myocardium distal to the ligation site and electrocardiogram (ECG) criteria (ST-segment elevation with upward convexity or T-wave inversion in lead II). The Sham group underwent the same surgical operation without LAD ligation. After 7 days, the echocardiography was performed to assess the infarct size, and the similar infarct degrees were used in this study. Finally, 21 rats were divided into three groups: Sham group (Sham, *n* = 6), MI alone group (MI, *n* = 8), and MI-exercise group (MI-Ex, *n* = 7).

### 2.3. Intervention

The exercise intervention protocol of patients with MI comprised three phases: Warm-up phase: 5-min low-intensity aerobic activities at 30%–40% heart rate reserve (HRR, corresponding to VO_2max_). Exercise training phase: ① Type: daily physical activity including but not limited to brisk walking, stair climbing, jogging, cycling, calisthenics, and playing ball; ② Duration: initial 10 min bouts progressively extended to 50 min based on individual exercise tolerance and symptom monitoring; ③ Frequency: 3 sessions weekly over a 12-week intervention period; ④ Intensity: MVPA prescription was determined by CPET and exercise risk stratification, starting at 40%–60% HRR (VO_2max_) and incrementally increasing to 70%–90% HRR (VO_2max_) with 10 min sustainability at target intensity, corresponding to rating of perceived exertion (RPE) 12–16 points and metabolic equivalent (MET) > 3.0. Cool-down phase: gradual reduction of workload within 5–10 min. The continuous single-lead Holter recorder coupled with CR manual was used to monitor exercise progression and adverse events. Intervention group participants were encouraged by telephone follow-up to increase their adherence to the scheduled exercise. Control group participants were provided on-time advice on standardized physical activity aligned with current guideline [19].

The exercise training protocol of rats was formulated as previously described [20]. The experiment started with 5 days of adaptive exercise training on a motorized rodent treadmill (Model XR-PT-10B; Shanghai Xinruan Technology, China) at 10 m/min (at an intensity of 40%–50% VO_2max_) for 30 min/day. Formal exercise training: rats performed daily 50 min-sessions of interval exercise training at treadmill speeds of 10 m/min for 10 min, then 25 m/min (at an intensity of 80%–90% VO_2max_) for 7 min, and 15 m/min *x* 3 min (at an intensity of 50%–60% VO_2max_) for 3 min, alternating exercise 3 group, 5 times per week for 4 weeks.

### 2.4. Echocardiography

Three-dimensional echocardiography (Philips EPIQ 7C, America) combined with software (TOMTEC-ARENA) was used to analyze left ventricular ejection fraction (LVEF) and the global longitudinal strain (GLS) of patients. The rats were anesthetized by 1%-2% isoflurane (RWD, Guangdong, China) until the heart rate was stabilized at 350–400 beats per minute. Two-dimensionally directed M mode echocardiography (VINNO6 LAB, China) was conducted to analyze left ventricular end-diastolic volume (LVEDV), left ventricular end-systolic volume (LVESV), stroke volume (SV), LVEF, and fractional shortening (FS).

### 2.5. CPET

CPET was performed on the cardiopulmonary function tester (COSMED Quark PE ergo, Italy) according to current recommendations [21], which was conducted by experienced medical staff. The test process is listed as follows: the resting phase when patients remain stationary for 3 min; the unloaded warm-up phase when patients begin to exercise for 3 min at a pace of 60 times per minute; the power load phase when exercises begin with an initial load and increase of workload developed according to age, height, and weight until it reached the maximum power load; and the cool down phase when patients kept pedaling for about 2-3 min with no power load [22]. The indicators included MET, peak oxygen consumption (VO_2_peak/kg), peak oxygen consumption as a percentage of predicted (VO_2_pred%), peak oxygen pulse (VO_2_HR), peak oxygen pulse as a percentage of predicted (VO_2_HRpred%), 1 min heart rate recovery (HRR1min), and peak power (Wmax).

### 2.6. SF-36 Quality of Life Scale

SF-36 was used to assess the quality of life of patients with MI by calculating the physical component summary (PCS) and mental component summary (MCS). Higher scores of SF-36 indicated better quality of life [22].

### 2.7. Cardiac Hemodynamic Measurement

Cardiac hemodynamic parameters were detected by the physiological polygraph system (BIOPAC MP160, Noldus, Nederland), consisting of a cardiac catheter, a pressure transducer, a signal receiver, and the ACqknowledge 5 PC analysis system. In brief, under surgical anesthesia, the rats were positioned in a supine position, and the catheter was connected to the pressure transducer inserted into the right carotid artery and advanced into left ventricular cavity, from which left ventricular systolic pressure (LVSP), left ventricular end-diastolic pressure (LVEDP), and positive and negative maximal left ventricular pressure rising rate (±dp/dt max) were recorded and measured by the ACqknowledge 5 PC analysis system.

### 2.8. Histological Staining and Analysis

The 4% formaldehyde-fixed cardiac tissue samples undergone gradient dehydration and paraffin embedding. Subsequently, the paraffin was sectioned into 5-μm-thick slices and stained with Masson's trichrome to analyze collagen volume fraction (CVF) according to the manufacturer's instructions. These data were performed via computerized image analysis with Image-Pro Plus 6.0 software (Media Cybernetics, Rockville, MD, USA).

### 2.9. Transmission Electron Microscope (TEM)

Left ventricular tissues were fixed with 2.5% glutaraldehyde in 0.1 M sodium phosphate (PH 7.4) overnight at 4°C. Samples were dehydrated through graded alcohols and were embedded in Epon Araldite following fixation in 1% OsO_4_ for 1 h. Ultrathin sections (60 nm) were produced by an ultra microtome (Leica UC7; Wetzlar, Germany) and stained with uranyl acetate and lead citrate. The specimens were visualized under a TEM (HT7800; HITACHI, China) and taken images. The mitochondrial flamengo score, aspect ratio, and area measurements were obtained using ImageJ (Version 1.42q, National Institutes of Health, Bethesda, Maryland, USA).

### 2.10. Biochemical Assay

Left ventricular samples (∼200 mg) were homogenized in ice-cold 0.1 mol/L phosphate buffer, PH 7.0, and centrifuged at 10, 000 g for 10 min at 4°C. The supernatant was assayed for measurement of total protein, malondialdehyde (MDA) and glutathione (GSH) levels by a microplate reader combined with the associated assay kit (Jiancheng Biotech, Nanjing, China).

### 2.11. Western Blot Analysis

Left ventricular tissues were extracted with radio-immunoprecipitation assay (RIPA) lysate buffer (R0010; Beijing Solarbio Science & Technology). The protein concentration was determined by a BCA protein assay kit (P0010; Beyotime). Proteins were separated through electrophoresis and transferred to PVDF membranes (Millipore, USA). The membranes were blocked with 5% nonfat milk for 2 h at room temperature and were incubated with appropriate primary antibodies at 4°C overnight. Membranes were washed by Tris-buffered saline containing 0.1% Tween 20 (TBST) and then subsequently probed with appropriate secondary antibodies at room temperature for 1 h. After washing with TBST three times, the protein bands were detected using an enhanced chemiluminescence detection solution (Affinity Biosciences, KF8003), and antibody detection was performed on a digitalized Bio-Rad ChemiDocTM MP Imaging system. Image J Software was used for intensity analysis.

### 2.12. Statistical Analysis

For clinical study, our study assumed that the change of LVEF in the exercise group was 4.82% according to the pretest result, and previous studies have shown that the standard deviation of LEVF was 8.82 [7]. A total of 70 patients were required to test for a power of at least 80% for group comparison with a loss-to-follow-up rate of 20% (α = 0.05). The categorical variable was presented as numbers (percentage) and compared with the Fisher's exact test at baseline. Normally distributed variables were expressed as mean ± SD and compared using the independent *t*-test for between-group differences, yet the paired *t*-test was used for within-group comparisons. Non-normally distributed variables were expressed as medians and interquartile ranges (IQRs) and compared using the Mann–Whitney *U* test for between-group differences and the Wilcoxon signed-rank test for within-group comparisons. All tests were 2-tailed, and *p* < 0.05 was taken as statistically significant. For animal research, all data were presented as mean ± standard error of mean. The unpaired *t*-test was used for normally distributed data during a comparison of 2 groups, while the Mann–Whitney *U* test was selected to analyze differences among nonparametric data. For comparisons between multiple groups, one-way ANOVA was performed. Statistical significance was set at *p* < 0.05. All statistical analyses were performed by SPSS software (version 25, SPSS Inc., Chicago, IL, USA) and GraphPad Prism software (version 8.3.0, La Jolla, CA, USA).

## 3. Results

### 3.1. Causal Association Between Six Physical Activities and MI

**Figure 1:**
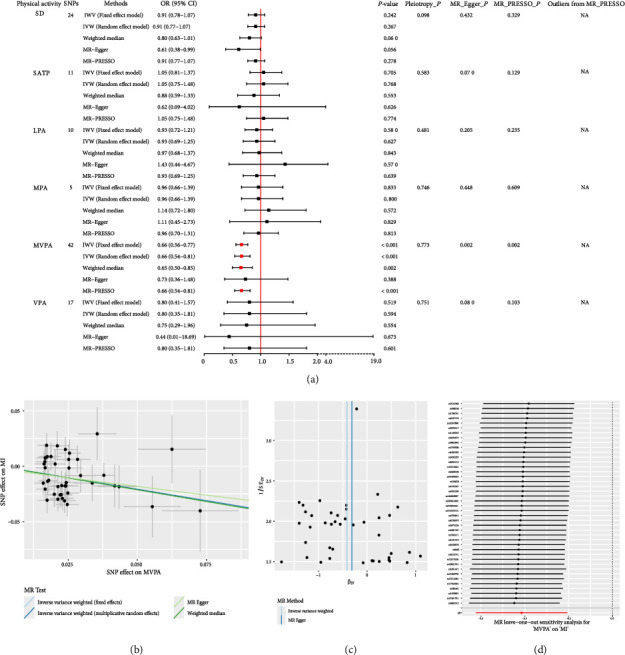
MR of six physical activities and MI risk. (a) MR estimates between six physical activities and MI. (b) Scatter plots of SNPs associated with MVPA and the risk of MI-based four different methods. The plot related the effect sizes of the SNP-MVPA association (*x*-axis, SD units) and the SNP-MI associations (*y*-axis, log [OR]) with 95% confidence intervals. (c) Funnel plot to assess the robustness between MVPA and MI. Scattering points represented the effect estimated using a single SNP as an instrumental variable. The vertical lines denoted the overall estimate obtained by the inverse variance weighted estimate and the MR-Egger regression. (d) The leave-one-out plots of sensitivity analysis between MVPA and MI.

Our TSMR results suggested that higher log odds of genetically predicted MVPA were associated with lower risk of MI (IVW OR = 0.66; 95% CI: 0.54–0.81; *p* < 0.001); the estimate from WM analysis was consistent with the IVW (Figures 1(a), 1(b)); there was no evidence of directional pleiotropy in the genetic instrument using the Egger intercept test (*p*=0.773). Additionally, there was no single SNP showing a significant impact on the MR estimation results based on leave-one-out analysis, with all significant estimates ranging from 0.54 to 0.81 (Figure 1(d)). However, both Cochran's *Q* test and *I*^2^-value supported the presence of heterogeneity for the analyses of MVPA with MI (*Q*-statistic, 71.64; *I*^2^ = 42.77%; *p*=0.002) (Figures 1(a), 1(c)). The heterogeneity was primarily attributable to multiancestry genetic architectures, as the meta-analyzed MVPA data originated from 51 studies with different ancestral composition (94.0% European, 2.1% African, 0.8% East Asian, 1.3% South Asian ancestries, and 1.9% Hispanic). Nonetheless, we observed that genetically predicated SD (IVW OR = 0.91; 95% CI: 0.78–1.07; *p*=0.242), SATP (IVW OR = 1.05; 95% CI: 0.81–1.37; *p*=0.705), MPA (IVW OR = 0.96; 95% CI: 0.66–1.39; *p*=0.833), and VPA (IVW OR = 0.80; 95% CI: 0.41–1.57; *p*=0.519) were no strong evidence for casual association with MI (Figure 1(a)). The other scatter plots, funnel plots, and leave-one-out plots can be found in Figures S3–S5.

### 3.2. Population Characteristics

70 patients with MI were finally enrolled in our study and divided into the control group (*n* = 35) and the exercise group (*n* = 35). During the 3-month follow-up period, attrition rates were 14.29% and 8.57% in the control and exercise groups, respectively. The study population had a mean age of 52.76 ± 9.31 years with predominant male representation (85.48%). Baseline characteristics including sex, age, type of MI, risk factors, laboratory indices, left ventricular function, cardiopulmonary function, and SF-36 scores showed no significant differences (all *p* > 0.05) (Table 1).

### 3.3. Beneficial Effects of Exercise-Based CR in Patients With MI

**Figure 2:**
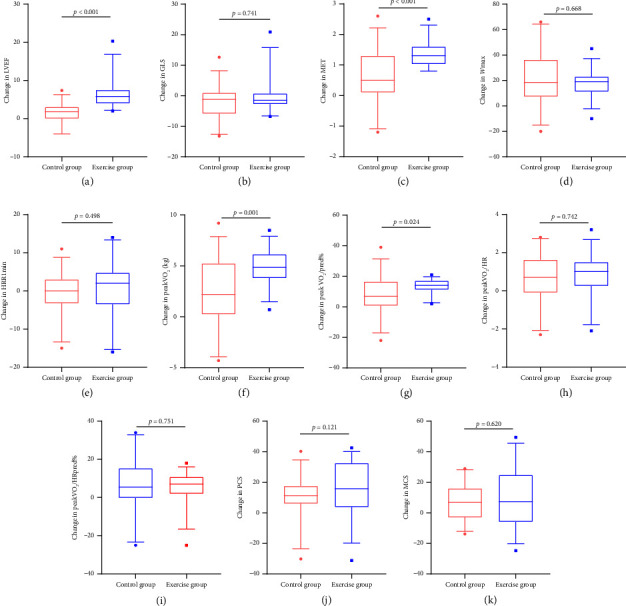
Beneficial effects in patients with MI from baseline to 3-month exercise-based CR. (a, b) Changes in left ventricular function. (c–i) Changes in cardiopulmonary function. (j, k) Changes in SF-36.

Following the 3-month intervention period, significant within-group differences were observed in multiple cardiopulmonary parameters, including LVEF, MET, Wmax, peakVO_2_/kg, peakVO_2_/pred%, peakVO_2_/HR, peakVO_2_/HRpred%, PCS, and MCS (all *p* < 0.05). Results of differential analysis from baseline to 3 months were shown in Table S3. Quantitative analysis revealed superior improvements in the exercise group compared to control, particularly in LVEF (1.31 ± 2.76 vs. 6.48 ± 3.84, *p* < 0.001), MET (0.63 ± 0.85 vs. 1.40 ± 0.44, *p* < 0.001), peakVO_2_/kg (2.41 ± 3.12 vs. 4.86 ± 1.83, *p*=0.001), and peakVO_2_/pred% (7.87 ± 12.37 vs. 13.50 ± 4.45, *p*=0.024). While a statistical difference in GLS was observed within the exercise group [−14.55 (−17.08, −12.23) vs. −16.45 (−17.88, −13.60), *p*=0.032], significant heterogeneity in the magnitude and direction of individual responses resulted in no significant difference in GLS improvement compared with the control group [−1.20 (−5.88, 0.95) vs. −1.45 (−2.75, 0.73), *p*=0.741]. Additionally, between-group comparisons showed no statistically significant differences in changes for GLS, Wmax, HRR1min, peakVO_2_/HR, peakVO_2_/HRpred%, PCS, and MCS from baseline to 3-month intervention (all *p* > 0.05) (Figure 2).

### 3.4. Compliance and Adverse Clinical Events

In the exercise group, 27 patients (77.14% completion rate) successfully completed the rehabilitation program, with all achieving the targeted exercise intensity. Within the control group, 2 patients who presented with chest tightness and pain were hospitalized and subsequently discharged after stabilization of symptoms. No patients experienced adverse clinical events in either group, including recurrent MI, malignant arrhythmia, heart failure, and death.

### 3.5. Cardioprotective Effects of HIIT in MI-Induced Left Ventricular Dysfunction

**Figure 3:**
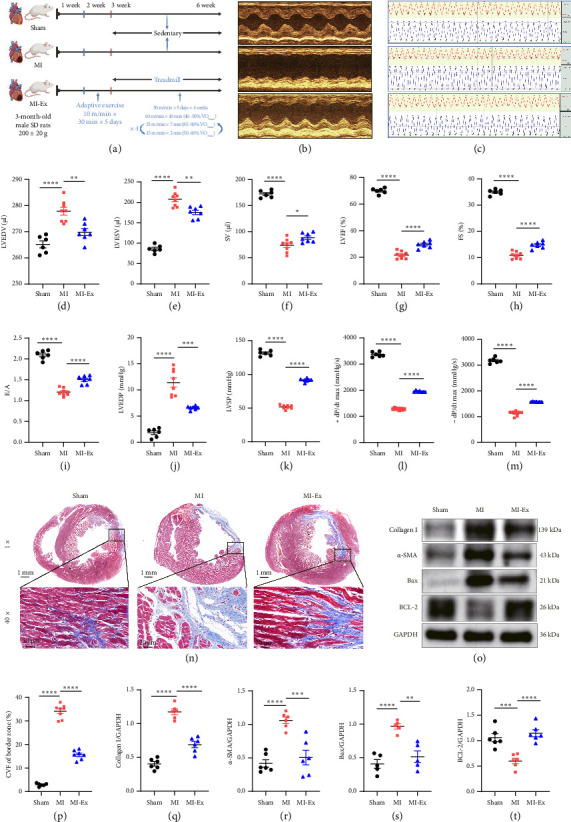
Beneficial effects of HIIT on MI-induced cardiac dysfunction and fibrosis. (a) A pattern diagram of the experimental protocol. (b, d–i) Echocardiography was applied to detect cardiac function of the rats in each group. (c, j–m) Physiological polygraph system was performed to detect cardiac hemodynamic parameters. (n, p) Cardiac fibrosis determined by Masson trichrome staining. (o, q–t) Western blot detected protein expression of cardiomyocyte apoptosis and fibrosis in the peri-infarcted area of myocardium. Symbol indicates ^∗^*p*  <  0.05, ^∗∗^*p* < 0.01, ^∗∗∗^*p* < 0.001, and ^∗∗∗∗^*p* < 0.0001.

To elucidate the protective role of HIIT in left ventricular dysfunction following MI, we established MI models in 3-month-old male SD rats, followed by a 4-week HIIT intervention protocol (Figure 3(a)). Echocardiographic assessment revealed that HIIT ameliorated MI-induced cardiac systolic and diastolic dysfunction, as evidenced by reduced LVEDV and LVESV, along with increased SV, LVEF, FS, and E/A ratio (all *p* < 0.05) (Figures 3(b), 3(d), 3(e), 3(f), 3(g), 3(h), and 3(i)). Hemodynamic evaluation corroborated these findings, showing decreased LVEDP and increased LVSP as well as improved ± dp/dp max (all *p* < 0.05) (Figures 3(c), 3(j), 3(k), 3(l), and 3(m)). Furthermore, HIIT substantially alleviated MI-induced fibrotic remodeling in myocardial border zones, concomitant with reversed cardiomyocyte apoptosis and fibrosis-related protein expressions (all *p* < 0.05) (Figures 3(n), 3(o), 3(p), 3(q), 3(r), 3(s), and 3(t)). Collectively, these findings established that HIIT exerted cardioprotective effects against MI-induced left ventricular dysfunction through functional improvement and pathological remodeling mitigation.

### 3.6. HIIT-Regulated Mitophagy and Oxidative Stress Signaling in MI Rats

**Figure 4:**
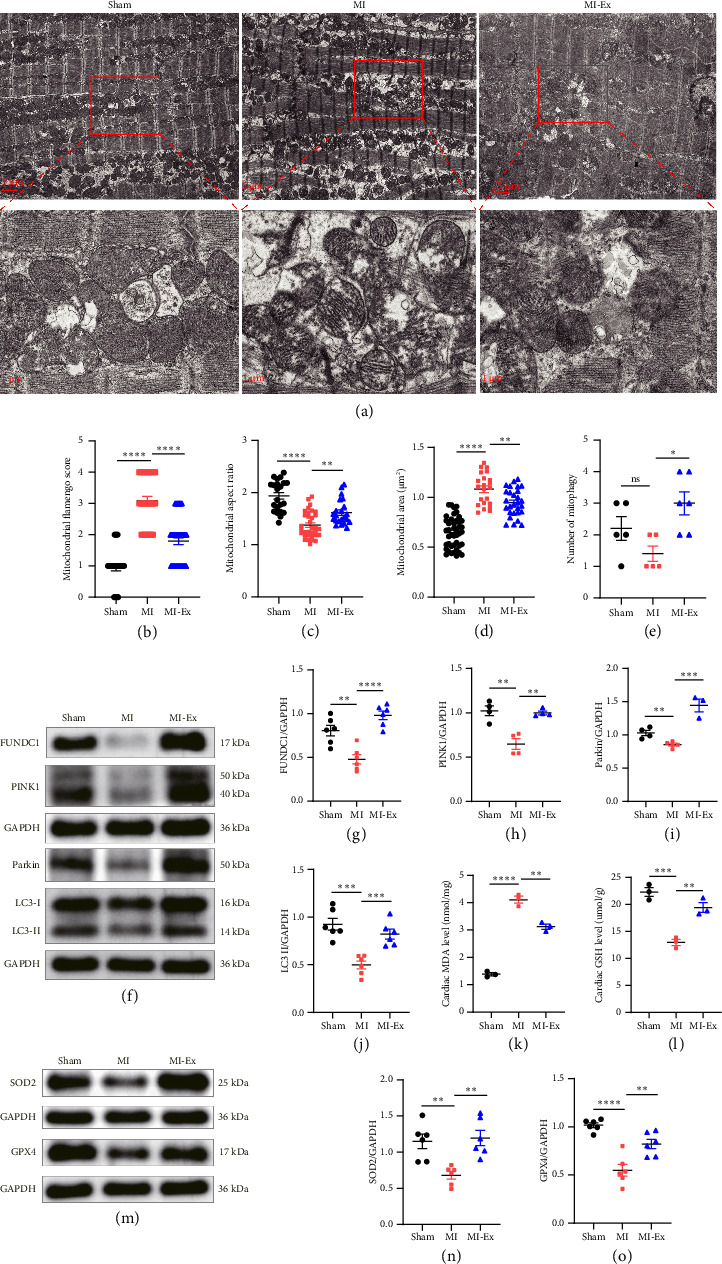
HIIT-regulated mitophagy and oxidative stress in MI rats. (a–e) TEM was employed to observe mitochondrial morphology and mitophagy. (f–j) Western results for mitophagy. (k–l) Biochemical assay detected myocardial oxidative levels. (m–o) Western results for oxidative stress. Symbol indicates ns: *p* > 0.05, ^∗^*p* < 0.05, ^∗∗^*p* < 0.01, ^∗∗∗^*p* < 0.001, and ^∗∗∗∗^*p* < 0.0001.

Previous investigations have established that mitochondrial architecture was directly affected by the disruption of quality control mechanisms in cardiovascular diseases. TEM analysis revealed severe mitochondrial disorganization in the MI heart, manifested by fragmented morphology (reduced aspect ratio), cristae disruption (elevated flamengo scores), and abnormal clustering (increased cross-sectional area). Concomitant with these ultrastructural damages, myocardial fibrosis and apoptosis were markedly increased after MI, resulting in left ventricular dysfunction. Notably, HIIT not only normalized mitochondrial architecture but also restored cardiac function (Figures 3(b), 3(c), 3(d), 3(e), 3(f), 3(g), 3(h), 3(i), 3(j), 3(k), 3(l), 3(m), 4(a), 4(b), 4(c), and 4(d)).

This functional recovery was mechanistically linked to HIIT-induced mitophagy activation. Compared with the MI-sedentary group, the number of mitophagy was increased in the HIIT group, and the expression of mitophagy-related proteins (FUNDC1, PINK1, Parkin, and LC3-II) were significantly upregulated, strongly correlating with cardiac fibrosis and apoptosis improvement (Figures 3(n), 3(o), 3(p), 3(q), 3(r), 3(s), 3(t), 4(a), 4(e), 4(f), 4(g), 4(h), 4(i), and 4(j)). Additionally, myocardial injury, mitochondrial disorganization, and mitophagy deficiency after MI would lead to decreased antioxidant enzyme activity and inability to effectively remove excessive ROS, reflecting by increased MDA, reduced GSH, superoxide dismutase 2 (SOD2), and glutathione peroxidase 4 (GPX4). However, 4-week HIIT intervention restored mitophagy levels, thereby improving antioxidant activity, evidenced by the reduced MDA level and increased GSH and antioxidant enzyme expressions (SOD2 and GPX4) compared with the MI-sedentary group (Figures 4(k), 4(l), 4(m), 4(n), and 4(o)). These findings supported HIIT-induced mitophagy formation to remove injured mitochondria, thereby reducing oxidative stress damage and improving left ventricular function after MI.

## 4. Discussion

### 4.1. Beneficial Effect of HIIT in Left Ventricular Function

In the present study, we applied TSMR analysis to evaluate the causal association between physical activity and MI. Our findings provided evidence to support the protective effect of MVPA on MI, suggesting that aerobic exercise was an effective prevention strategy for MI risk. Similarly, a prospective cohort study in 130,000 individuals from 17 low-income, middle-income, and high-income countries confirmed that higher recreational and nonrecreational physical activity was associated with a lower risk of mortality and cardiovascular disease events [23]. A meta-analysis of 33 prospective cohort studies also reported that higher levels of physical activity were associated with a lower risk of coronary heart disease [24]. A study of the China population demonstrated that sports-related physical activity was related with a lower risk of onset of MI than inactivity [25]. Nevertheless, there was no evidence of association of genetically predicted SD, SATP, LPA, MPA, and VPA with MI, which was inconsistent with previous findings [26–28]. Zhuang et al. [27] reported that self-reported VPA was associated with a lower risk for MI. The difference between our findings and previous results may be primarily attributed to heterogeneity in the definitions and assessment methodologies of PA, particularly variations in intensity categorization criteria and measurement precision across different cohorts.

Our study showed that a 3-month exercise training improved left ventricular systolic function and cardiopulmonary fitness. In heart failure with reduced ejection fraction, one of the most common complications of MI, HIIT was superior to control for improvement of LVEF during 2-3 months [29]. Additionally, previous studies demonstrated that participation in exercise-based CR by subjects with coronary heart disease receiving contemporary medical management significantly mitigated cardiovascular mortality, recurrent cardiac events, and hospitalizations [30]. A systematic review and meta-analysis confirmed that HIIT enhanced cardiopulmonary fitness in post-MI patients compared with MICT and routine physical activity [7]. However, the changes in GLS, other cardiopulmonary functions, and SF-36 at 3 months were not apparent differences between the exercise-based CR group and the control group, and this might attribute to shorter intervention duration. Eser et al. [31] reported that both GLS and cardiopulmonary fitness in patients with MI were significantly improved at 1-year follow-up in the exercise-based CR. Dibben et al. [32] revealed that exercise-based CR may slightly increase health-related quality of life (HRQoL) up to 12 months of follow-up.

### 4.2. Regulation of HIIT in Mitochondrial Function and Mitophagy

Progressive left ventricular remodeling post-MI is the pivotal driver of transition to heart failure and significantly reduced survival [33]. Therefore, alleviating maladaptive cardiac remodeling is the first-line strategy to treat reversible causes. In this study, we confirmed that HIIT enhanced left ventricular systolic and diastolic function and attenuated MI-induced myocardial fibrosis, which was consistent with previous research [34]. In pathological conditions such as myocardial ischemia hypoxia and inflammatory infiltration, mitochondrial structural and functional disorders exacerbated left ventricular remodeling after MI [35]. Additionally, imbalance between mitochondrial division and fission promoted fragmented mitochondria formation, resulting in cardiac metabolic alteration and excessive oxidative stress [36]. Ding et al. [37] revealed that mitochondrial division inhibitor 1 (Mdivi-1) alleviated myocardial fibrosis post-MI via inhibiting Drp1-activated mitochondrial fission and Hmox1-associated oxidative stress. Notably, this study confirmed that HIIT played a cardioprotective role by attenuating mitochondrial damage and fragmentation production. And studies also reported that HIIT maintained the balance of the mitochondrial network to enable cells to survive normally through regulating mitochondrial dynamics and mitophagy [38, 39].

Next, we found that MI rats performed a downregulation of the mitophagy signaling-related proteins FUNDCI, PINK1, Parkin, and LC3-II levels, which was in line with the reported study [40]. Mitophagy maintained mitochondrial homeostasis and dynamics by selectively degrading and evacuating the damaged or aged mitochondria [11]. For instance, knockdown or knockout of FUNDCI destroyed the formation of mitochondrial-endoplasmic reticulum-associated membranes, damaged to mitochondrial function, and inhibited interaction with LC3 motif against mitophagy formation, resulting in cardiac dysfunction [41]. Furthermore, the activity of FUNDCI-mediated mitophagy was related to the intensity and duration of exercise training [42]. Under the same exercise intensity, mitophagy activation of 4-week exercise was higher than that in the 2-week exercise group; under the same duration of exercise, the high-intensity exercise group was higher than that in the moderate-intensity exercise group [42]. Our data showed that 4-week HIIT upregulated the mitophagy signaling biomarkers FUNDCI, PINK1, Parkin, and LC3-II in MI rats. Therefore, mitophagy represented a promising yet underexplored monitoring and prognostic indicator for exercise-based CR. Its clinical validation warrants in-depth investigation through noninvasive methodologies, particularly via the development of echocardiography contrast agents incorporating molecular probes specific to mitophagy pathways.

### 4.3. Inhibition of HIIT in Oxidative Stress

We demonstrated that MI rats increased cardiac oxidative stress with accumulation of MDA, decreased levels of GSH, and reduced activity of the antioxidant enzymes SOD2 and GPX4, consistent with another study [43]. Mitochondrial damage and insufficient mitophagy promoted the formation of excessive oxidative products, resulting in cardiac dysfunction post-MI [44]. For instance, the deficiency of SOD2 led to an accumulation of ROS and subsequent overproduction of 4-hydroxynonenal within MI-induced mitochondrial structural abnormalities [45]. Current studies reported that exercise training significantly decelerated the production of oxidative products and increased the activity of antioxidant enzymes [8]. Moreover, our findings demonstrated that inhibition of oxidative stress by exercise training may be the potential mechanism of action of mitophagy. Therefore, targeting mitophagy may be the therapeutic strategies for the prevention and treatment of MI.

### 4.4. Limitations and Future Perspectives

Some limitations should be considered in this study. First, the long-term effects of exercise-based CR on GLS and quality of life remained unclear. Consequently, large-scale and multicenter clinical trials are still needed to support the cardioprotective role of HIIT, particularly the MI population with high risk of exercise. Second, we still could not completely rule out the role of other lifestyle factors such as diet in the progression of MI. Third, the mechanism underlying exercise-based CR remained unelucidated, particularly PINK1/Parkin or FUNDC1-dependent mitophagy, mitochondrial quality control, mitochondria-related oxidative stress, as well as their mechanisms of interaction in myocardial fibrosis. Finally, mitophagy pathways still needed to be explored in the clinical study, such as utilizing human-induced pluripotent stem cell-derived cardiomyocytes to model mitophagy mechanisms under physiological and pathological conditions and exploring novel noninvasive imaging techniques such as echocardiography coupled with mitophagy-targeted contrast agents to dynamically detect levels of mitochondrial autophagy in injured myocardium of post-MI patients.

## 5. Conclusions

This study provided evidence for the protective effect of MVPA on the initiation of MI. Our clinical data demonstrated 3-month exercise-based CR significant improved left ventricular systolic function and cardiopulmonary function, though no significant short-term improvement in quality of life was observed in patients with MI. HIIT prevented the progression of left ventricular dysfunction, increased cardiac mitophagy, and reduced oxidative stress post-MI. Furthermore, the molecular mechanism of exercise-mediated mitophagy in CR deserved further study.

## Figures and Tables

**Table 1:** Baseline characteristics of study participants.

	Control group (*n* = 30)	Exercise group (*n* = 32)	*p*
Male sex, *n* (%)	24 (80.00)	29 (90.63)	0.235
Age (year)	52.17 ± 10.18	53.31 ± 8.54	0.184
Type of MI, *n* (%)			0.184
STEMI	10 (33.33)	16 (50.00)	
NSTEMI	20 (66.67)	16 (50.00)	
Description of coronary lesions, *n* (%)			0.122
Single-vessel disease	10 (33.33)	15 (46.88)	
Double-vessel disease	15 (50.00)	8 (25.00)	
Triple-vessel disease	5 (16.67)	9 (28.13)	
Risk factors			
BMI (kg/m^2^)	24.43 ± 2.80	24.99 ± 2.69	0.356
Smoking, *n* (%)	20 (66.67)	25 (78.13)	0.312
Hypertension, *n* (%)	14 (46.67)	12 (37.50)	0.465
Hyperlipidemia, *n* (%)	15 (50.00)	19 (59.38)	0.459
Diabetes mellitus/impaired glucose tolerance, *n* (%)	9 (30.00)	9 (28.13)	0.871
Laboratory test			
Total cholesterol (mmol/L)	3.36 (2.70, 3.78)	3.42 (3.09, 4.29)	0.257
Triglyceride (mmol/L)	1.58 (1.15, 2.83)	1.68 (1.20, 2.25)	0.647
Low density lipoprotein (mmol/L)	1.62 ± 0.82	1.98 ± 0.81	0.151
Creatinine (μmol/L)	87.55 (80.28, 100.90)	88.75 (77.23, 103.25)	0.921
Uric acid (μmol/L)	370.40 ± 110.80	418.75 ± 97.16	0.072
Echocardiography			
LVEF (%)	49.40 ± 6.83	49.19 ± 5.43	0.892
GLS (%)	−14.50 (−17.75, −12.03)	−14.55 (−17.08, −12.23)	0.827
CPET			
MET	5.34 ± 0.96	5.00 ± 0.86	0.155
Wmax (watt)	120.80 ± 32.26	130.47 ± 31.34	0.757
HRR1min (beats)	22.23 ± 6.82	19.56 ± 7.05	0.695
peakVO_2_/kg (mL/kg/min)	18.69 ± 3.39	18.50 ± 4.25	0.306
peakVO_2_/pred%	62.67 ± 11.12	60.59 ± 11.50	0.578
peakVO_2_/HR (mL/beat)	10.17 ± 1.83	9.67 ± 2.04	0.307
peakVO_2_/HRpred%	80.73 ± 12.85	75.50 ± 13.85	0.480
SF-36			
PCS	57.79 ± 13.10	61.41 ± 13.77	0.295
MCS	62.51 ± 18.41	67.00 ± 13.95	0.282

*Note:* NSTEMI, non-ST-segment elevation myocardial infarction; STEMI, ST-segment elevation myocardial infarction.

Abbreviation: BMI, body mass index.

## Data Availability

The data that support the findings of this study are available in the supporting information of this article.

## Supporting Information

Table S1: Detailed information of GWAS data.

Table S2: Genetic variants used as instrumental variables for the physical activity pattern.

Table S3: Outcomes of exercise and quality of life after 3 months.

Figure S1: Study design and three assumptions of TSMR analysis.

Figure S2: Flowchart of the data collection, processing, and analysis procedures of this study.

Figure S3: Scatter plot of the association between physical activity and myocardial infarction.

Figure S4: Funnel plot to assess the robustness between physical activity and myocardial infarction.

Figure S5: The sensitive analyses of TSMR analysis.
