# Supplementary material for: Exercise-Based Cardiac Rehabilitation Improves Left Ventricular Dysfunction, Mitophagy, and Oxidative Stress Postmyocardial Infarction
Source: Cardiol Res Pract. 2025 Jul 23;2025:7778063. doi: 10.1155/crp/7778063 (PMC12310327; doi:10.1155/crp/7778063)
Supplement: Supporting Information — Additional supporting information can be found online in the Supporting Information section. [file 7778063.f1.docx]

**Supplementary Tables**

| Table S1: Detailed information for the GWAS data. | | | | | | | |
| --- | --- | --- | --- | --- | --- | --- | --- |
|  | **GWAS ID** | **Data source** | **Sample size** | **Number of SNPs** | **PMID** | **Year** | **population** |
| Exposure | | | | | | | European |
| SD | GCST006914 | Doherty A et al. | 101,307 | 9,770,928 | 30531941 | 2018 |  |
| SATP | GCST90061428 | Qi G et al. | 88,411 | 8,489,912 | 35043453 | 2022 |  |
| LPA | GCST90061421 | Qi G et al. | 88,411 | 8,489,912 | 35043453 | 2022 |  |
| MPA | GCST006915 | Doherty A et al. | 101,307 | 9,770,928 | 30531941 | 2018 |  |
| MVPA | GCST90104342 | Wang Z et al. | 703,901 | 49,915,722 | 36071172 | 2022 |  |
| VPA | GCST006098 | Klimentidis YC et al. | 261,055 | 11,834,614 | 29899525 | 2018 |  |
| Outcome | | | | | | |  |
| MI | GCST011364 | Hartiala JA et al. | 395,795 | 10,290,368 | 33532862 | 2021 |  |
| SD, sleep duration; SATP, sedentary-to-activity transition probability; LPA: light-intensity physical activity; MPA, moderate-intensity physical activity; MVPA, moderate-to-vigorous intensity physical activity; VPA, vigorous-intensity physical activity; MI, myocardial infarction. | | | | | | | |

| **Table S2:** Genetic variants used as instrumental variables for physical activity pattern. | | | | | | | | | | | |
| --- | --- | --- | --- | --- | --- | --- | --- | --- | --- | --- | --- |
| **SNP** | **EA** | **OA** | **Associated with different physical activity** | | | | | **Associated with myocardial infarction** | | | |
|  |  |  | **EAF** | **Beta** | **SE** | ***P*** | **F-statistics** | **EAF** | **Beta** | **SE** | **P** |
| **Sleep duration by Doherty A et al.** | | | | | | | |  |  |  |  |
| rs10823730 | T | C | 0.59511 | 0.027268008 | 0.004688103 | 1.00E-09 | 33.83 | 0.581311 | -0.005962214 | 0.010993359 | 0.59 |
| rs113851554 | G | T | 0.943193 | 0.089661111 | 0.010292586 | 1.10E-17 | 75.89 | 0.94435 | -0.020091947 | 0.024288518 | 0.41 |
| rs115066682 | T | C | 0.975557 | 0.080518008 | 0.015433831 | 8.50E-08 | 27.22 | 0.975666 | 0.02338314 | 0.036256549 | 0.52 |
| rs11672103 | C | T | 0.446783 | -0.029679693 | 0.004644291 | 1.20E-10 | 40.84 | 0.45833 | -0.000738161 | 0.010894148 | 0.95 |
| rs1198575 | T | C | 0.190057 | -0.036997318 | 0.005867529 | 1.20E-10 | 39.76 | 0.188579 | 0.014864564 | 0.013767844 | 0.28 |
| rs12190064 | G | A | 0.718039 | 0.028771839 | 0.005131149 | 2.70E-08 | 31.44 | 0.72207 | -0.005835913 | 0.011995429 | 0.63 |
| rs13002630 | T | A | 0.707293 | 0.027913793 | 0.00507795 | 7.30E-09 | 30.22 | 0.708847 | -0.006284754 | 0.011854295 | 0.60 |
| rs2006810 | T | C | 0.604456 | -0.027645019 | 0.004703659 | 1.50E-08 | 34.54 | 0.601561 | -0.015687775 | 0.011015496 | 0.15 |
| rs2416963 | C | T | 0.589103 | 0.029741571 | 0.004668889 | 1.80E-09 | 40.58 | 0.58199 | -0.019509864 | 0.010903355 | 0.07 |
| rs2693333 | T | C | 0.785808 | 0.03513659 | 0.005681054 | 8.20E-10 | 38.25 | 0.792269 | -0.019574792 | 0.013445165 | 0.15 |
| rs2741348 | T | G | 0.415478 | -0.021781992 | 0.004698946 | 9.30E-08 | 21.49 | 0.424301 | -0.016663986 | 0.010968647 | 0.13 |
| rs3006964 | T | C | 0.952719 | -0.053986398 | 0.010811475 | 2.10E-07 | 24.93 | 0.954564 | -0.018819083 | 0.02571343 | 0.46 |
| rs329124 | A | G | 0.576647 | 0.02374023 | 0.004655345 | 3.90E-07 | 26.01 | 0.577841 | 0.00799588 | 0.010874166 | 0.46 |
| rs336606 | T | C | 0.276028 | -0.025796935 | 0.005145421 | 1.60E-07 | 25.14 | 0.276527 | -0.017510257 | 0.012057054 | 0.15 |
| rs6128763 | T | C | 0.575355 | 0.022882375 | 0.004680881 | 4.10E-07 | 23.90 | 0.558244 | -0.005669396 | 0.010997837 | 0.61 |
| rs62158170 | A | G | 0.783008 | -0.051014559 | 0.005585709 | 4.10E-19 | 83.41 | 0.785605 | 0.021810377 | 0.01312408 | 0.10 |
| rs62508246 | G | C | 0.756013 | 0.030598467 | 0.005406207 | 1.80E-08 | 32.03 | 0.764459 | -0.010939541 | 0.012790458 | 0.39 |
| rs6439701 | T | C | 0.605221 | -0.026764176 | 0.004718678 | 6.00E-09 | 32.17 | 0.598247 | -0.006473128 | 0.010991316 | 0.56 |
| rs6931466 | A | G | 0.746915 | 0.037899042 | 0.005310057 | 1.50E-13 | 50.94 | 0.750285 | 0.012261857 | 0.012439988 | 0.32 |
| rs7166130 | A | T | 0.897783 | 0.038205939 | 0.007657088 | 1.20E-07 | 24.90 | 0.883924 | -0.013966322 | 0.017289895 | 0.42 |
| rs72694315 | C | T | 0.983492 | -0.095650192 | 0.018057433 | 2.00E-07 | 28.06 | 0.984065 | 0.033350472 | 0.042789343 | 0.44 |
| rs72810611 | T | G | 0.908178 | -0.038385441 | 0.007983276 | 4.20E-07 | 23.12 | 0.907502 | 0.012216575 | 0.018624692 | 0.51 |
| rs73192215 | G | A | 0.839272 | 0.032190996 | 0.006263506 | 6.10E-08 | 26.41 | 0.836535 | 0.019323728 | 0.014518152 | 0.18 |
| rs73606355 | G | A | 0.955288 | 0.056626245 | 0.011168084 | 6.40E-08 | 25.71 | 0.951336 | -0.039488663 | 0.025351206 | 0.12 |
| **Sedentary-to-activity transition probability by Qi G et al.** | | | | | | | |  |  |  |  |
| rs12021614 | C | T | 0.262225 | -0.0270374 | 0.00533453 | 4.01E-07 | 25.69 | 0.271443 | 0.012055879 | 0.012421041 | 0.33 |
| rs12325879 | T | C | 0.707813 | 0.0260665 | 0.00500963 | 1.96E-07 | 27.07 | 0.688646 | 0.027426683 | 0.011865266 | 0.02 |
| rs12610468 | C | T | 0.460993 | 0.0253977 | 0.00456558 | 2.65E-08 | 30.95 | 0.475728 | -0.003366168 | 0.01085841 | 0.76 |
| rs159963 | C | A | 0.421988 | 0.0242588 | 0.00466175 | 1.95E-07 | 27.08 | 0.42437 | -0.011632925 | 0.01095902 | 0.29 |
| rs2316767 | T | C | 0.785792 | -0.0282647 | 0.0056097 | 4.69E-07 | 25.39 | 0.788745 | -0.014782229 | 0.013311055 | 0.27 |
| rs3104551 | G | A | 0.570404 | -0.0277949 | 0.00460956 | 1.64E-09 | 36.36 | 0.557428 | -0.019228884 | 0.010906854 | 0.08 |
| rs528657806 | T | C | 0.884131 | -0.0364223 | 0.00723188 | 4.74E-07 | 25.36 | 0.882471 | -0.000947164 | 0.017209547 | 0.96 |
| rs5831317 | T | A | 0.812044 | -0.0307211 | 0.00592509 | 2.16E-07 | 26.88 | 0.803886 | 0.00556733 | 0.013712068 | 0.68 |
| rs61779075 | T | C | 0.600304 | -0.0235347 | 0.00465386 | 4.26E-07 | 25.57 | 0.613166 | 0.015622735 | 0.01107589 | 0.16 |
| rs75721495 | G | A | 0.95759 | -0.0574428 | 0.0113675 | 4.34E-07 | 25.54 | 0.956106 | 0.031755265 | 0.026826409 | 0.24 |
| rs987633 | C | A | 0.413075 | 0.0241035 | 0.00464123 | 2.07E-07 | 26.97 | 0.420582 | 0.011866301 | 0.010972481 | 0.28 |
| **Light-intensity physical activity by Qi G et al.** | | | | | | | |  |  |  |  |
| rs10166518 | C | T | 0.338717 | 0.0257545 | 0.00496515 | 2.14E-07 | 26.91 | 0.333799 | -0.001568612 | 0.011468367 | 0.89 |
| rs11179465 | G | T | 0.975086 | -0.0775534 | 0.0149425 | 2.10E-07 | 26.94 | 0.975834 | -0.000476612 | 0.035207909 | 0.99 |
| rs11672103 | C | T | 0.44591 | 0.026204 | 0.00473327 | 3.09E-08 | 30.65 | 0.45833 | -0.000738161 | 0.010894148 | 0.95 |
| rs12021614 | C | T | 0.262225 | -0.027801 | 0.00545936 | 3.54E-07 | 25.93 | 0.271443 | 0.012055879 | 0.012421041 | 0.33 |
| rs1268539 | C | A | 0.581337 | -0.029715 | 0.00472658 | 3.24E-10 | 39.52 | 0.575418 | -0.018020752 | 0.010907749 | 0.10 |
| rs12717867 | A | G | 0.546649 | 0.0290392 | 0.004694 | 6.15E-10 | 38.27 | 0.540669 | -0.015054673 | 0.010785898 | 0.16 |
| rs1359741 | G | A | 0.53091 | -0.0239573 | 0.00467467 | 2.98E-07 | 26.26 | 0.522052 | -0.003787219 | 0.010812988 | 0.73 |
| rs647347 | A | G | 0.574432 | 0.0243079 | 0.00476278 | 3.33E-07 | 26.05 | 0.571001 | -0.024571541 | 0.010915333 | 0.02 |
| rs74800845 | G | A | 0.944843 | -0.0520209 | 0.0102196 | 3.57E-07 | 25.91 | 0.94582 | 0.00785956 | 0.023953524 | 0.74 |
| rs9878906 | A | C | 0.16135 | 0.0377487 | 0.00634377 | 2.67E-09 | 35.41 | 0.175406 | 0.014901533 | 0.014559179 | 0.31 |
| **Moderate-intensity physical activity by Doherty A et al.** | | | | | | | |  |  |  |  |
| rs10849145 | T | C | 0.825346 | 0.035157181 | 0.006206117 | 1.10E-08 | 32.09 | 0.826821 | -0.021772988 | 0.014349256 | 0.13 |
| rs147568733 | G | A | 0.973357 | -0.078163032 | 0.015059601 | 3.60E-07 | 26.94 | 0.974222 | -0.016441384 | 0.035386461 | 0.64 |
| rs17244293 | G | A | 0.947093 | 0.055724468 | 0.010709096 | 4.20E-07 | 27.08 | 0.948573 | 0.008991457 | 0.025046186 | 0.72 |
| rs183671 | T | G | 0.022462 | 0.081054787 | 0.016148404 | 5.30E-08 | 25.19 | 0.0584916 | -0.002345719 | 0.032056675 | 0.94 |
| rs62092069 | G | A | 0.627375 | -0.025993085 | 0.004825106 | 9.70E-08 | 29.02 | 0.632907 | -0.004637688 | 0.011150697 | 0.68 |
| **Moderate-to-vigorous intensity physical activity by Wang Z et al.** | | | | | | | |  |  |  |  |
| rs10210242 | A | G | 0.4989 | 0.016 | 0.0032 | 3.63E-07 | 25.00 | 0.50284 | -0.015251387 | 0.010730261 | 0.16 |
| rs10821761 | T | C | 0.388 | 0.0175 | 0.0032 | 6.71E-08 | 29.91 | 0.401279 | 0.008692566 | 0.010936463 | 0.43 |
| rs1160545 | T | C | 0.4025 | 0.0249 | 0.0041 | 1.73E-09 | 36.88 | 0.392553 | -0.024242341 | 0.011038892 | 0.03 |
| rs12357890 | A | G | 0.4431 | 0.0225 | 0.0041 | 4.77E-08 | 30.12 | 0.448164 | -0.025701592 | 0.010869296 | 0.02 |
| rs12973258 | T | C | 0.8199 | 0.0252 | 0.0047 | 6.79E-08 | 28.75 | 0.813025 | 0.00614001 | 0.013786791 | 0.66 |
| rs13201721 | T | C | 0.7364 | 0.0255 | 0.004 | 1.83E-10 | 40.64 | 0.735221 | 0.011807474 | 0.0123035 | 0.34 |
| rs13262776 | T | G | 0.0567 | -0.0355 | 0.0069 | 2.57E-07 | 26.47 | 0.054951 | -0.029296966 | 0.023865228 | 0.22 |
| rs144949097 | A | G | 0.0227 | 0.0727 | 0.0138 | 1.43E-07 | 27.75 | 0.021349 | -0.040278151 | 0.037043518 | 0.28 |
| rs1531519 | T | C | 0.3797 | 0.0166 | 0.0033 | 3.73E-07 | 25.30 | 0.392203 | 0.004150702 | 0.011058762 | 0.71 |
| rs1625595 | T | C | 0.4748 | -0.0213 | 0.0032 | 1.90E-11 | 44.31 | 0.471934 | 0.018384067 | 0.010760011 | 0.09 |
| rs1691471 | T | C | 0.3759 | 0.0379 | 0.0042 | 1.73E-19 | 81.43 | 0.369625 | -0.008164972 | 0.011170259 | 0.46 |
| rs17762954 | T | C | 0.2036 | 0.0211 | 0.0041 | 2.47E-07 | 26.48 | 0.216454 | 0.01842683 | 0.013070823 | 0.16 |
| rs1788761 | A | G | 0.5026 | -0.0213 | 0.0041 | 1.55E-07 | 26.99 | 0.49555 | 0.029795678 | 0.010783155 | 0.01 |
| rs2012741 | A | C | 0.606 | 0.0169 | 0.0032 | 1.70E-07 | 27.89 | 0.595628 | 0.007625792 | 0.010917824 | 0.48 |
| rs2074117 | T | G | 0.3522 | 0.0174 | 0.0033 | 1.97E-07 | 27.80 | 0.319064 | -0.030688994 | 0.011636479 | 0.01 |
| rs2281767 | T | C | 0.4874 | -0.0163 | 0.0032 | 2.80E-07 | 25.95 | 0.487144 | -0.002084698 | 0.010812037 | 0.85 |
| rs2634673 | C | G | 0.3355 | 0.0221 | 0.0044 | 4.90E-07 | 25.23 | 0.32932 | -0.025939586 | 0.011468087 | 0.02 |
| rs2668196 | A | T | 0.1908 | -0.0227 | 0.004 | 2.09E-08 | 32.21 | 0.195311 | 0.017470601 | 0.013587362 | 0.20 |
| rs2675124 | A | G | 0.204 | -0.0203 | 0.0039 | 2.45E-07 | 27.09 | 0.203116 | -0.00202644 | 0.013441415 | 0.88 |
| rs3095255 | A | C | 0.3502 | 0.0239 | 0.0044 | 5.55E-08 | 29.50 | 0.356306 | -0.017712961 | 0.01136048 | 0.12 |
| rs3109891 | T | C | 0.5969 | 0.0172 | 0.0032 | 8.96E-08 | 28.89 | 0.601036 | 0.018895737 | 0.010971586 | 0.09 |
| rs336620 | C | G | 0.3016 | 0.0243 | 0.0044 | 4.05E-08 | 30.50 | 0.302475 | -0.014780018 | 0.01172763 | 0.21 |
| rs370935521 | T | C | 0.0926 | 0.0434 | 0.0075 | 8.25E-09 | 33.49 | 0.087953 | -0.018460245 | 0.019744275 | 0.35 |
| rs374576715 | A | G | 0.0816 | 0.0418 | 0.0077 | 5.10E-08 | 29.47 | 0.083479 | -0.017758942 | 0.019445776 | 0.36 |
| rs3799000 | T | C | 0.472 | -0.0167 | 0.0032 | 1.15E-07 | 27.24 | 0.470375 | 0.020701596 | 0.0107742 | 0.06 |
| rs3818978 | A | T | 0.3774 | -0.0167 | 0.0033 | 2.83E-07 | 25.61 | 0.391897 | -0.007527588 | 0.010998256 | 0.49 |
| rs385301 | T | C | 0.2637 | -0.0284 | 0.0047 | 1.60E-09 | 36.51 | 0.264819 | -0.006191924 | 0.012171433 | 0.61 |
| rs4352559 | T | C | 0.4964 | 0.018 | 0.0032 | 1.65E-08 | 31.64 | 0.483021 | -0.012742518 | 0.010884605 | 0.24 |
| rs4730073 | A | C | 0.6458 | 0.0167 | 0.0033 | 4.90E-07 | 25.61 | 0.647282 | -0.00137187 | 0.011245318 | 0.90 |
| rs4743347 | T | C | 0.692 | 0.0176 | 0.0034 | 2.77E-07 | 26.80 | 0.674565 | -0.013541717 | 0.011442676 | 0.24 |
| rs4790841 | T | C | 0.1556 | 0.0295 | 0.0056 | 1.58E-07 | 27.75 | 0.150201 | -0.008442566 | 0.015094273 | 0.58 |
| rs4865512 | A | G | 0.6124 | 0.024 | 0.0042 | 7.68E-09 | 32.65 | 0.610857 | 0.015161972 | 0.011034162 | 0.17 |
| rs529031892 | A | C | 0.1296 | -0.0337 | 0.0065 | 2.13E-07 | 26.88 | 0.131496 | 0.015210752 | 0.016605383 | 0.36 |
| rs568546 | T | C | 0.5206 | 0.0237 | 0.0041 | 5.89E-09 | 33.41 | 0.523431 | -0.031297132 | 0.010777418 | 0.004 |
| rs57012093 | T | C | 0.0292 | -0.0625 | 0.0122 | 2.99E-07 | 26.24 | 0.032744 | -0.015345001 | 0.030598879 | 0.62 |
| rs6427178 | A | G | 0.5318 | 0.0229 | 0.0041 | 1.71E-08 | 31.20 | 0.517904 | -0.029054326 | 0.010820713 | 0.01 |
| rs6663 | A | G | 0.2369 | -0.0188 | 0.0037 | 3.72E-07 | 25.82 | 0.245024 | -0.008799137 | 0.012492406 | 0.48 |
| rs75499504 | A | G | 0.9592 | 0.0554 | 0.0103 | 8.43E-08 | 28.93 | 0.96093 | -0.036548444 | 0.027782862 | 0.19 |
| rs7613360 | T | C | 0.3963 | -0.0247 | 0.0042 | 2.77E-09 | 34.59 | 0.386602 | 0.034560591 | 0.011059154 | 0.002 |
| rs7920171 | A | G | 0.2311 | -0.0253 | 0.0048 | 1.62E-07 | 27.78 | 0.233345 | 0.001616259 | 0.012727293 | 0.90 |
| rs8094118 | T | G | 0.7382 | 0.0242 | 0.0046 | 1.82E-07 | 27.68 | 0.74258 | -0.017806378 | 0.01233241 | 0.15 |
| rs9903845 | A | C | 0.3104 | -0.02 | 0.0034 | 6.05E-09 | 34.60 | 0.321733 | 0.025348799 | 0.011513173 | 0.03 |
| **Vigorous-intensity physical activity by Klimentidis YC et al.** | | | | | | | |  |  |  |  |
| rs1248860 | G | A | 0.48444 | -0.00976972 | 0.00131429 | 1.10E-13 | 55.26 | 0.481998 | 0.015486946 | 0.010750831 | 0.15 |
| rs13182417 | G | A | 0.961422 | -0.0177665 | 0.00341296 | 1.90E-07 | 27.10 | 0.962251 | 0.000736765 | 0.02814142 | 0.98 |
| rs13243553 | G | A | 0.607979 | 0.00874851 | 0.00134959 | 9.00E-11 | 42.02 | 0.601931 | -0.009406295 | 0.011015132 | 0.39 |
| rs1461584 | A | G | 0.794414 | -0.0087476 | 0.0016315 | 8.20E-08 | 28.75 | 0.793567 | -0.024247714 | 0.013306018 | 0.07 |
| rs2663648 | C | T | 0.883587 | -0.0106654 | 0.00205249 | 2.00E-07 | 27.00 | 0.862109 | 0.000273537 | 0.016353564 | 0.99 |
| rs2764261 | A | G | 0.374311 | 0.00912633 | 0.00136054 | 2.00E-11 | 45.00 | 0.387329 | -0.011868148 | 0.011144847 | 0.29 |
| rs328902 | C | T | 0.685492 | -0.00878858 | 0.0014168 | 5.50E-10 | 38.48 | 0.68986 | 0.027008823 | 0.011611684 | 0.02 |
| rs3781411 | C | T | 0.876382 | 0.0125792 | 0.00199721 | 3.00E-10 | 39.67 | 0.877636 | -0.003483485 | 0.016370328 | 0.83 |
| rs58242881 | G | A | 0.792593 | 0.00871339 | 0.00162764 | 8.60E-08 | 28.66 | 0.796798 | 0.006498875 | 0.013400527 | 0.63 |
| rs61866271 | G | C | 0.983421 | 0.0277644 | 0.00514764 | 6.90E-08 | 29.09 | 0.98415 | 0.021400689 | 0.042906605 | 0.62 |
| rs61897072 | C | T | 0.805009 | 0.0085382 | 0.0016596 | 2.70E-07 | 26.47 | 0.807775 | -0.000633454 | 0.013650807 | 0.96 |
| rs6667222 | A | C | 0.748143 | 0.00873051 | 0.00151739 | 8.70E-09 | 33.10 | 0.744179 | 0.01266939 | 0.012413289 | 0.31 |
| rs6689056 | G | A | 0.674264 | 0.00766132 | 0.00141772 | 6.50E-08 | 29.20 | 0.675416 | 0.009035899 | 0.011599873 | 0.44 |
| rs72924609 | G | A | 0.875421 | 0.010498 | 0.00199776 | 1.50E-07 | 27.61 | 0.877776 | -0.018923499 | 0.016472141 | 0.25 |
| rs72928932 | T | G | 0.917797 | -0.012992 | 0.00241492 | 7.50E-08 | 28.94 | 0.92086 | 0.013269244 | 0.020061246 | 0.51 |
| rs9276758 | G | A | 0.688307 | 0.00803189 | 0.00141706 | 1.40E-08 | 32.13 | 0.68793 | 0.028598993 | 0.011554704 | 0.01 |
| rs9387216 | C | A | 0.748303 | -0.00790523 | 0.00151917 | 2.00E-07 | 27.08 | 0.737385 | 0.012699475 | 0.01232656 | 0.30 |

| **Table S3:** Outcomes of exercise and quality of life after 3 months. | | | | | | | | | |
| --- | --- | --- | --- | --- | --- | --- | --- | --- | --- |
|  | | **Control group (n=30)** | | | ***P1*** | **Exercise group (n=32)** | | ***P2*** | ***P*** |
|  | | **Baseline** | | **3-month** |  | **Baseline** | **3-month** |  |  |
| Echocardiography | | | | | | | | | |
| LVEF (%) | 49.40 ± 6.83 | | 50.60 ± 6.91 | | 0.021 | 49.19 ± 5.43 | 55.69 ± 7.29 | < 0.001 | < 0.001 |
| GLS (%) | -14.50 (-17.75, -12.03) | | -15.15 (-19.83, -12.80) | | 0.090 | -14.55 (-17.08, -12.23) | -16.45 (-17.88, -13.60) | 0.032 | 0.741 |
| CPET | | | | | | | | | |
| MET | 5.34 ± 0.96 | | 5.96 ± 1.23 | | < 0.001 | 5.00 ± 0.86 | 6.4 ± 0.82 | < 0.001 | < 0.001 |
| Wmax (watt) | 120.80 ± 32.26 | | 141.23 ± 35.83 | | < 0.001 | 130.47 ± 31.34 | 148.94 ± 32.16 | < 0.001 | 0.668 |
| HRR1min (beats) | 22.23 ± 6.82 | | 21.53 ± 6.92 | | 0.521 | 19.56 ± 7.05 | 20.03 ± 6.17 | 0.724 | 0.498 |
| peakVO2/kg (mL/kg/min) | 18.69 ± 3.39 | | 21.10 ± 4.53 | | < 0.001 | 18.50 ± 4.25 | 23.37 ± 0.77 | < 0.001 | 0.001 |
| peakVO2/pred% | 62.67 ± 11.12 | | 70.53 ± 12.34 | | 0.002 | 60.59 ± 11.50 | 74.09 ± 8.68 | < 0.001 | 0.024 |
| peakVO2/HR (mL/beat) | 10.17 ± 1.83 | | 9.57 ± 2.25 | | 0.019 | 9.67 ± 2.04 | 10.38 ± 2.19 | 0.003 | 0.742 |
| peakVO2/HRpred% | 80.73 ± 12.85 | | 86.47 ± 16.78 | | 0.028 | 75.50 ± 13.85 | 80.31 ± 13.58 | 0.004 | 0.751 |
| SF-36 | | | | | | | | | |
| PCS | 57.79 ± 13.10 | | 68.23 ± 11.47 | | < 0.001 | 61.41 ± 13.77 | 78.02 ± 13.24 | < 0.001 | 0.121 |
| MCS | 62.51 ± 18.41 | | 69.64 ± 16.31 | | 0.006 | 67.00 ± 13.95 | 76.14 ± 14.05 | 0.011 | 0.620 |
| P1 and P2 showed the difference within the control and exercise group before and after 3 months, respectively; P showed the difference of changes between control group and exercise group. LVEF, left ventricular ejection fraction; GLS, global longitudinal strain; MET, metabolic equivalent; PCS, physical component summary; MCS, mental component summary. | | | | | | | | | |

**Supplementary Figures**


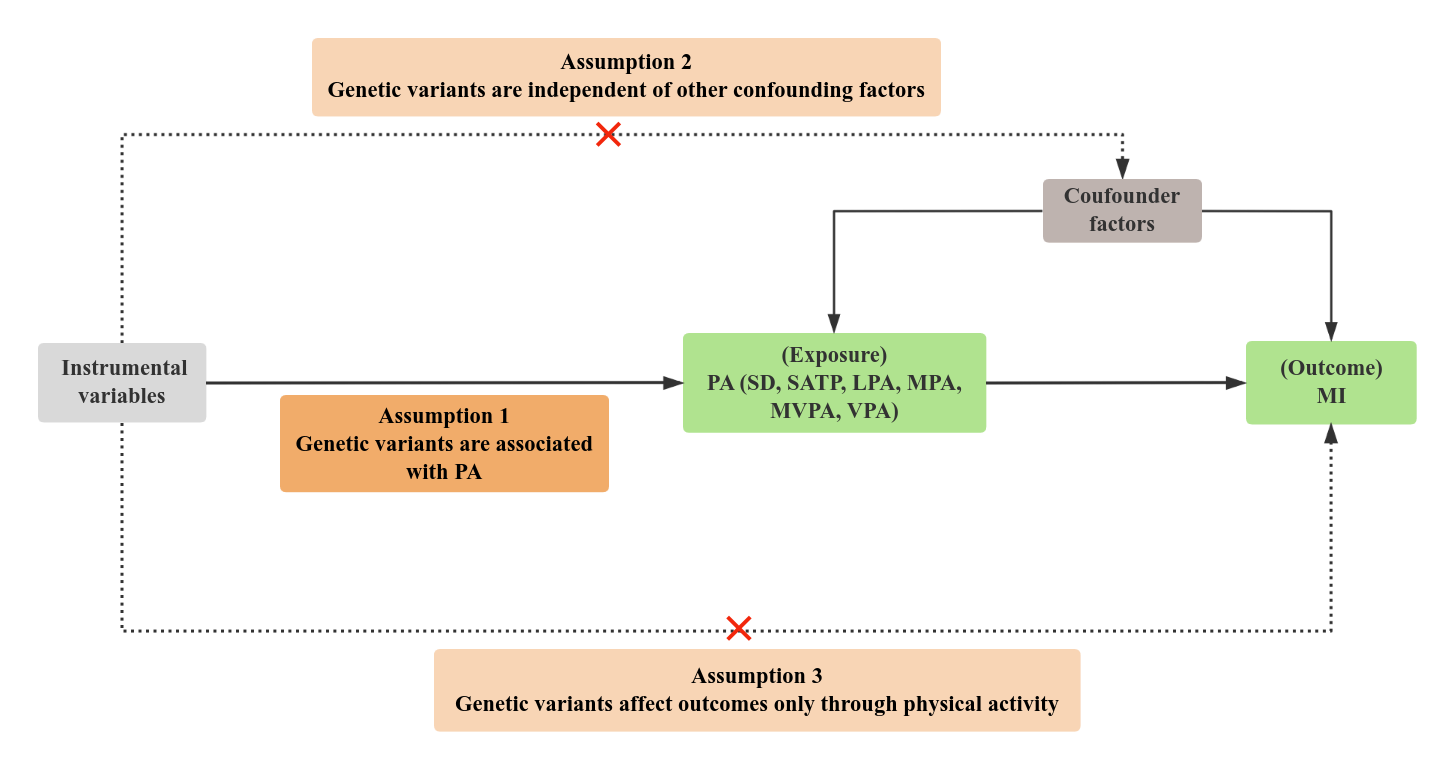


**Figure S1:** Study design and 3 assumptions of TSMR analysis. By comparing the PA of individuals with different genetic variants, we may draw causal inference about the effects of PA on MI , provided 3 key assumptions are met that address (1) each genetic variant should be associated with PA, (2) the genetic variant is independent of any confounders, and (3) the association between each genetic variant and MI only occurs via the PA. SD, sleep duration; SATP, sedentary-to-activity transition probability; LPA, light-intensity physical activity; MPA, moderate-intensity physical activity; MVPA, moderate-to-vigorous intensity physical activity; VPA, vigorous-intensity physical activity.


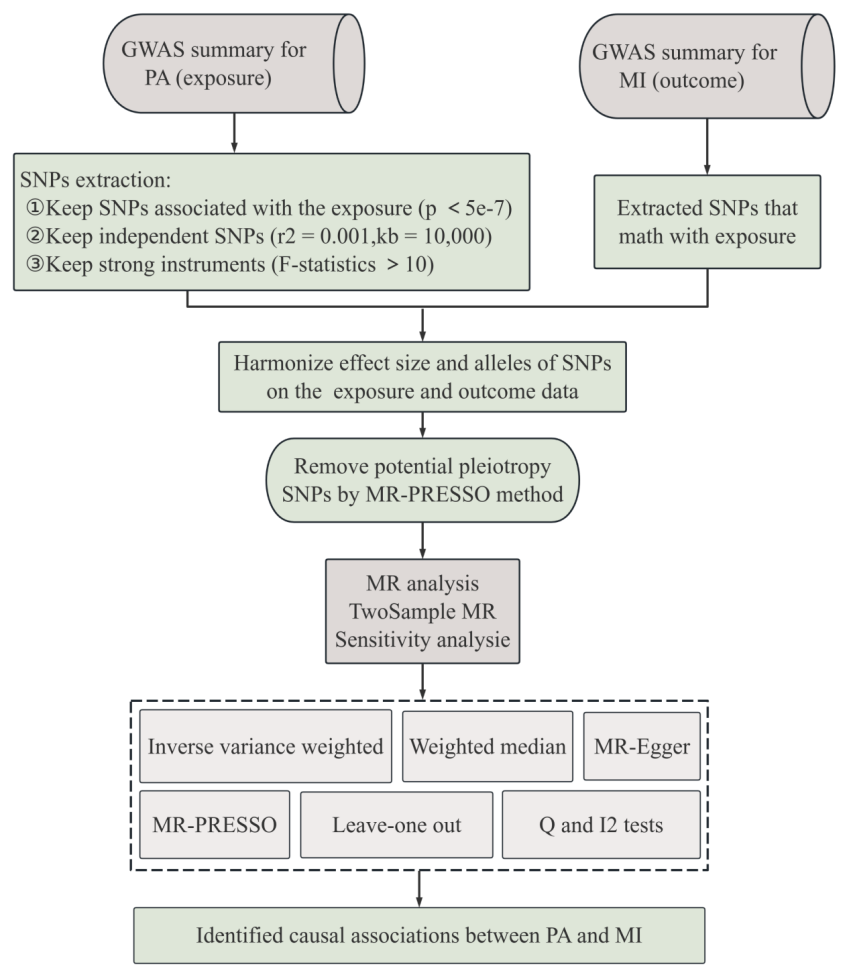


**Figure S2:** Flowchart of the data collection, processing, and analysis procedures of this study.


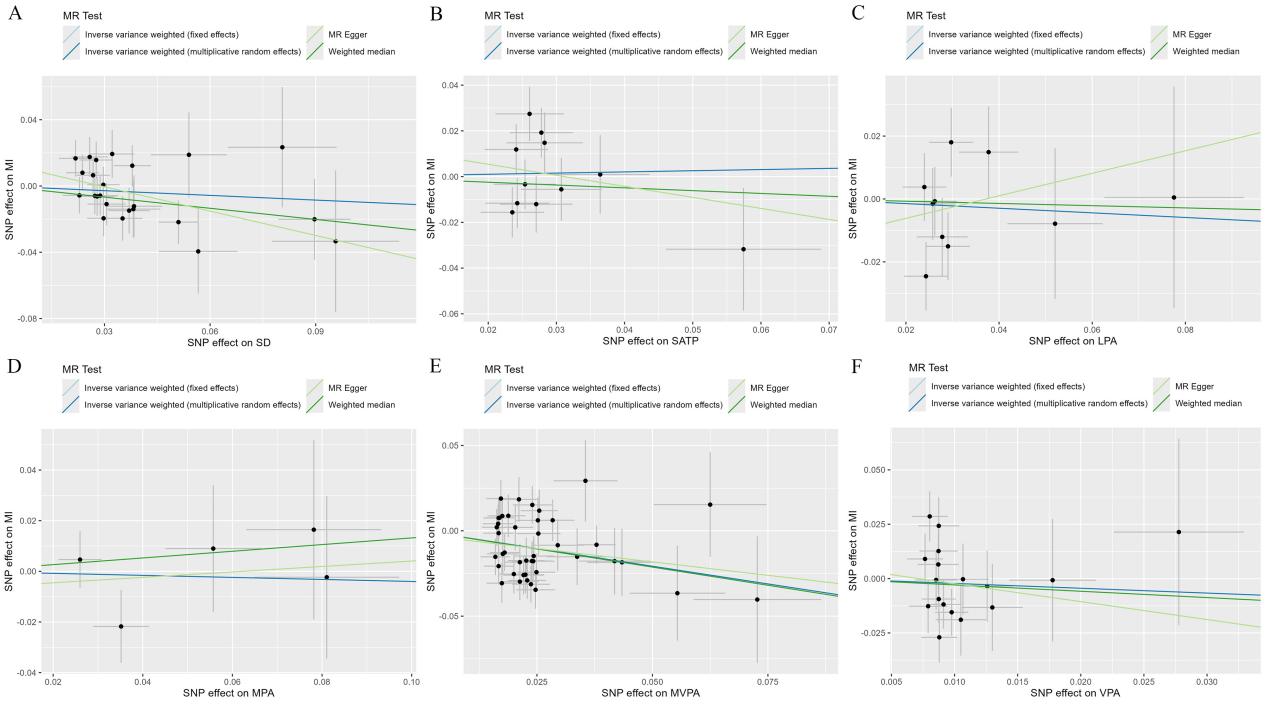


**[Figure S](https://europepmc.org/articles/PMC9349767/figure/jmv28008-fig-0003/)3:** Scatter plot of the association between physical activity and myocardial infarction. A, SD; B, SATP; C, LPA; D, MPA; E, MVPA; F, VPA.


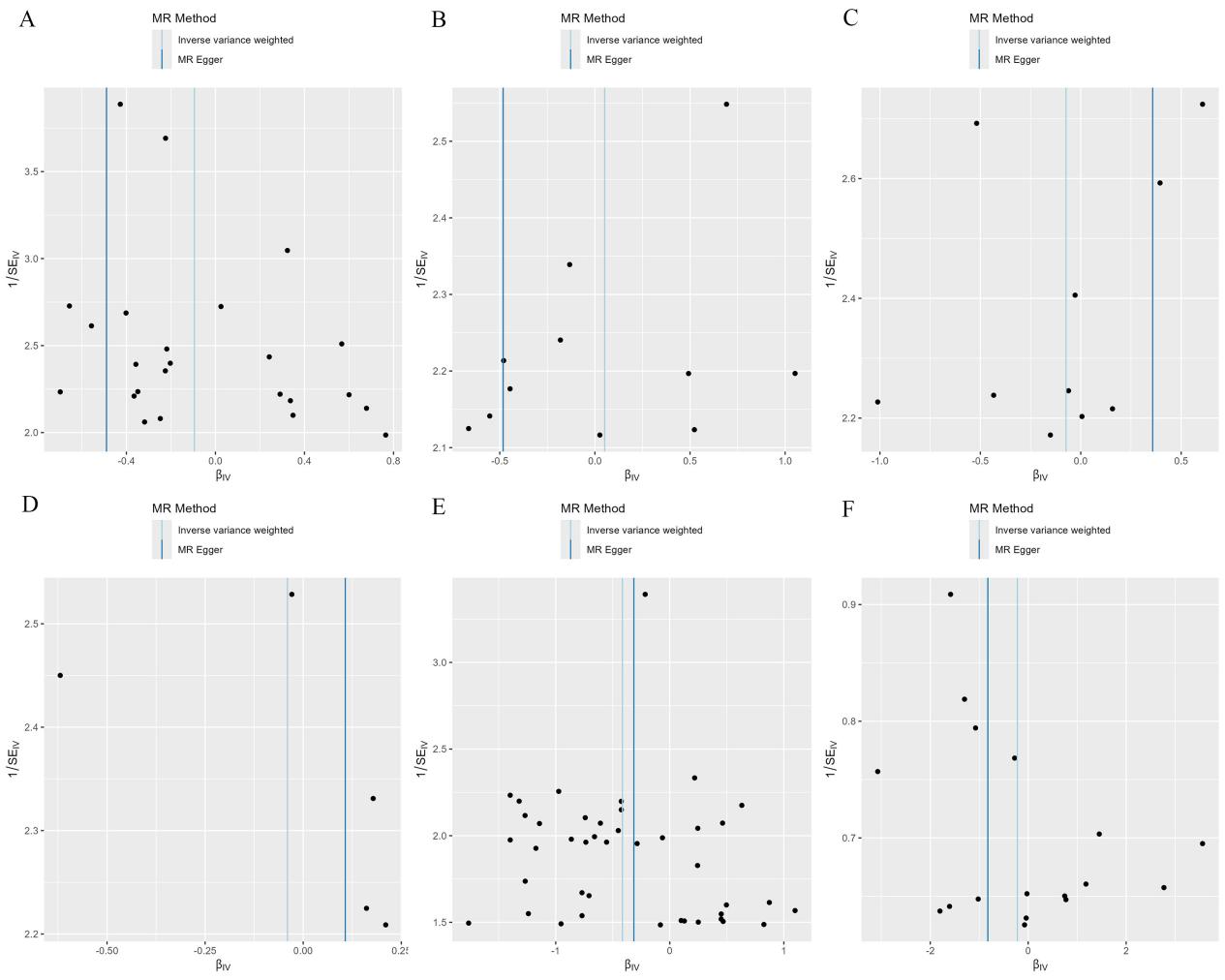


**[Figure S](https://europepmc.org/articles/PMC9349767/figure/jmv28008-fig-0003/)4:** Funnel plot to assess the robustness between physical activity and myocardial infarction. A, SD; B, SATP; C, LPA; D, MPA; E, MVPA; F, VPA.


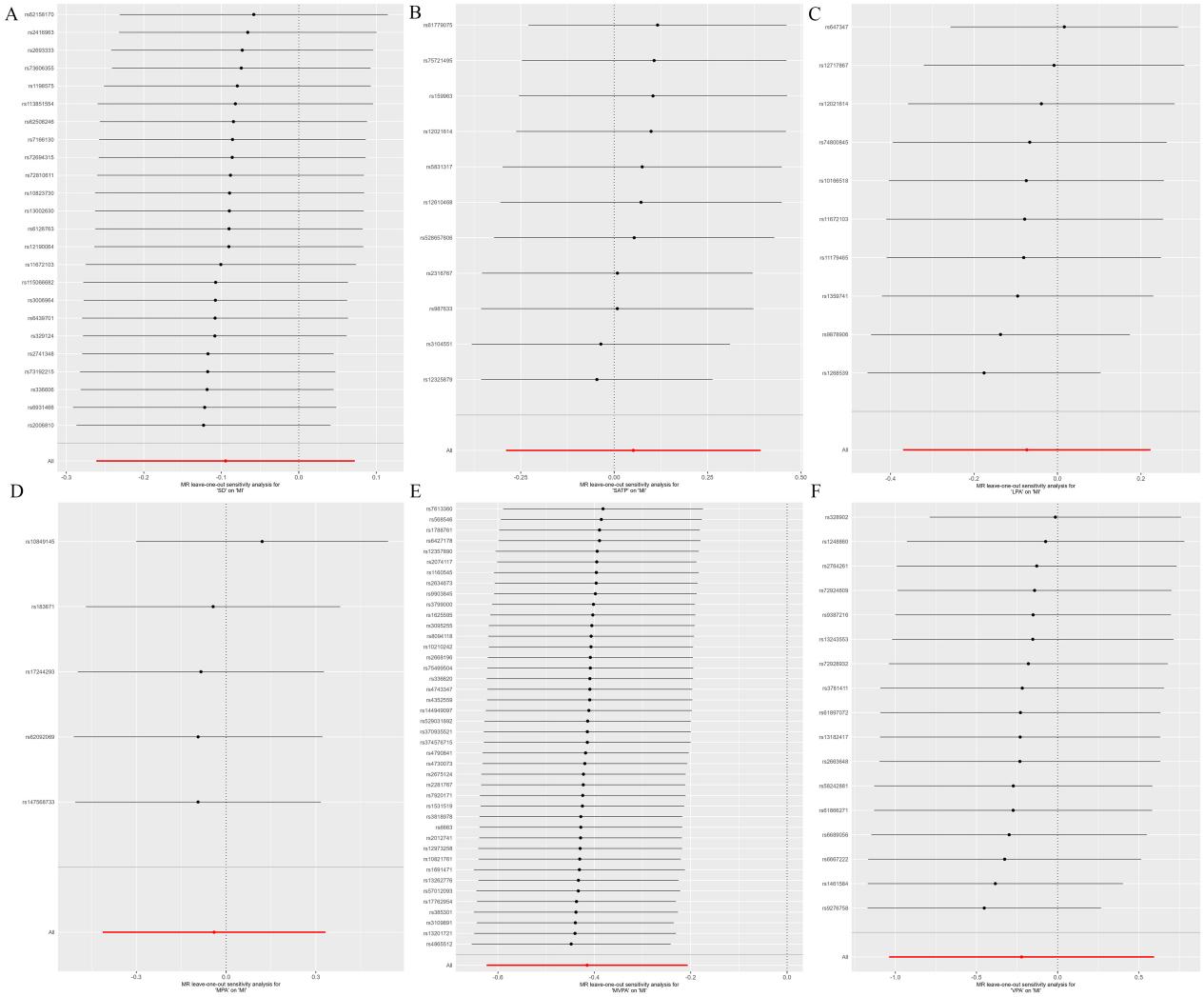


**Figure S5:** The sensitive analyses of MR analyses for MI based on SD (A), SATP (B), LPA (C), MPA (D), MVPA(E), and VPA (F).
